# The Manganese–Bone Connection: Investigating the Role of Manganese in Bone Health

**DOI:** 10.3390/jcm13164679

**Published:** 2024-08-09

**Authors:** Gulaim Taskozhina, Gulnara Batyrova, Gulmira Umarova, Zhamilya Issanguzhina, Nurgul Kereyeva

**Affiliations:** 1 Department of Laboratory Diagnostics, West Kazakhstan Marat Ospanov Medical University, 68 Maresyev Street, Aktobe 030019, Kazakhstan; 2 Department of Evidence-Based Medicine and Scientific Management, West Kazakhstan Marat Ospanov Medical University, 68 Maresyev Street, Aktobe 030019, Kazakhstan; gulmira.um80@gmail.com; 3 Department of Children Disease No. 2, West Kazakhstan Marat Ospanov Medical University, 68 Maresyev Street, Aktobe 030019, Kazakhstan; gamilia0452@gmail.com; 4 Department of Oncology, West Kazakhstan Marat Ospanov Medical University, 68 Maresyev Street, Aktobe 030019, Kazakhstan; nrgl230777@gmail.com

**Keywords:** manganese, bone, bone health, bone mass, bone metabolism

## Abstract

The complex relationship between trace elements and skeletal health has received increasing attention in the scientific community. Among these minerals, manganese (Mn) has emerged as a key element affecting bone metabolism and integrity. This review examines the multifaceted role of Mn in bone health, including its effects on bone regeneration, mineralization, and overall skeletal strength. This review article is based on a synthesis of experimental models, epidemiologic studies, and clinical trials of the mechanisms of the effect of Mn on bone metabolism. Current research data show that Mn is actively involved in the processes of bone remodeling by modulating the activity of osteoblasts and osteoclasts, as well as the main cells that regulate bone formation and resorption. Mn ions have a profound effect on bone mineralization and density by intricately regulating signaling pathways and enzymatic reactions in these cells. Additionally, Mn superoxide dismutase (MnSOD), located in bone mitochondria, plays a crucial role in osteoclast differentiation and function, protecting osteoclasts from oxidative damage. Understanding the nuances of Mn’s interaction with bone is essential for optimizing bone strategies, potentially preventing and managing skeletal diseases. Key findings include the stimulation of osteoblast proliferation and differentiation, the inhibition of osteoclastogenesis, and the preservation of bone mass through the RANK/RANKL/OPG pathway. These results underscore the importance of Mn in maintaining bone health and highlight the need for further research into its therapeutic potential.

## 1. Introduction

The main support function in the human body is provided by the structural integrity of bones [1]. Bone is a dynamic tissue that is constantly being formed and resorbed [2], and the presence of various minerals and nutrients in these bones has a significant impact on overall health. Of these minerals, often overshadowed by their better-known counterparts such as calcium and phosphorus [3,4], manganese (Mn) has stood out for its importance in maintaining bone integrity and function [5,6]. Recent research has led to a deeper understanding of the complex interactions between Mn and bone biology [7]. Research has not only identified the presence of Mn in various components of bone tissue, suggesting its possible involvement in bone matrix formation and remodeling [8], but has also highlighted adverse effects on skeletal development and integrity in studies of Mn deficiency, indicating the important role of Mn in maintaining optimal bone health [9]. This review article summarizes the results of various studies examining the relationship between Mn and bone metabolism. To review the current state of knowledge on Mn in bone physiology and pathology, it is important to contextualize information on the status of Mn in bone health and bone homeostasis and disorders.

## 2. Historical Overview: Early Discoveries on the Relationship between Mn and Bone Health

In 1774, Mn was isolated by Gahn [10]. Mn is named after the Greek word for magic. Mn is 0.01% of the earth’s mass. While Mn is the twelfth most abundant element (Lakni and Venugopal 1977), due to its propensity to oxidize and form insoluble nodules in the ocean, its abundance in seawater may be only parts per billion [11]. An important mineral source is pyrolucite, MnO_2_. There are other ores, such as manganite, MnO(OH); brounite Mn_2_O_3_; and rhodochrosite, MnCO_3_. Mn(OH)_2_ in a saline solution is easily oxidized when exposed to air [12]. The element Mn can also exist in many states, I, II, III, IV, VI or VII, in organic and inorganic compounds. The most stable form is Mn(II) [13].

In 1931, it was mentioned that Mn is an important nutrient [14], and it is necessary for the bone growth of rats and mice. It was reported and proven by Wilgus et al. (1939) that Mn can prevent a skeletal abnormality called perosis in chickens [15]. A number of studies have shown that it accumulates in high amounts in the mitochondria of bone tissue. In bone development, it was found that Mn is also necessary for the functioning of key enzymes, that is, it has a high ability to protect cells from damage by free radicals [16].

Also, Mn affects hormones involved in bone regeneration and enzyme products in bone metabolism, respectively, and its function is related to bone. Stimulating effects of Mn on bone are shown when increasing the intracellular calcium release and activating one or more enzymes [17]. Concentrations of Mn in human bone under normal conditions of 1.7–3.0 mg/kg wet weight have also been reported [18].

Although Mn has many oxidation states, the two most common forms found in the human body are Mn^2+^ and Mn^3+^ [19]. Basically, since Mn^2+^ in the body is chemically more stable than Mn^3+^, Mn can be incorporated into metalloenzymes, mainly as Mn^2+^ [20]. Mn^2+^ can be oxidized to Mn^3+^ by ceruloplasmin and transported in circulation by transferrin (Tf) [21]. Importantly, redox between Mn^2+^ and Mn^3+^ provides a “double-edged sword” effect in cellular homeostasis [22]. Mn catalyzes superoxide (O_2_^−^) to hydrogen peroxide (H_2_O_2_) through the Mn^2+^/Mn^3+^ cycle; its function as a cofactor of cellular Mn superoxide dismutase (MnSOD) detoxifies free radicals in mitochondria, thus preventing oxidative stress [23]. Mn is present in the reactive center of bone composition and many enzymes.

## 3. The Influence of Mn Metabolism on Bone Mass

**Figure 1:**
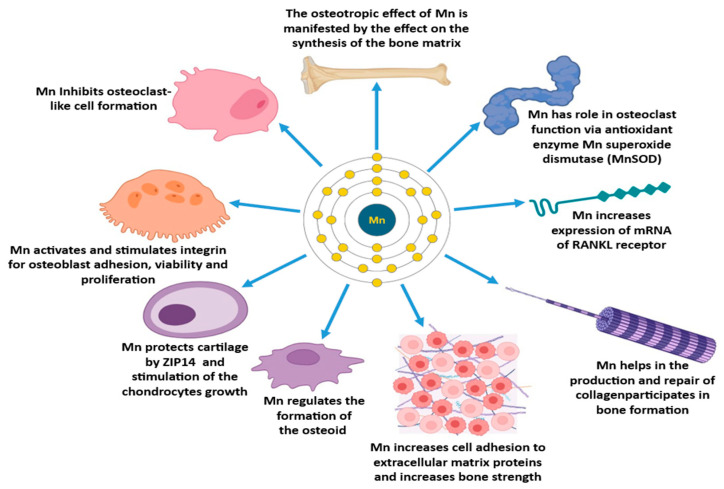
The role of manganese (Mn) in bone cellular and molecular functions. The trace element Mn, with its various biochemical and physiological effects, participates in the synthesis of bone matrix, the inhibition of the formation of osteoclast-like cells, antioxidant function with the enzyme Mn superoxide dismutase (MnSOD), and mRNA expression of RANKL receptors; it also contributes to cell adhesion with extracellular matrix proteins, regulating osteoid formation. It also protects cartilage and stimulates chondrocyte growth through ZIP14. This is important for its integrin-activating functions, which contribute to the adhesion, integrity, and proliferation of osteoblasts.

Mn is an essential element for humans and animals [24], necessary for normal processes in the bone [25] (Figure 1). The mineral density and micro-architectural integrity of bone tissue determines its strength [26]. For bone mass development, the osteotropic effect is important. The osteotropic effect of Mn is manifested through the effect of the synthesis of the bone matrix [27]. Bone matrix integrity is important in bone strength and plastic deformation [28]. The bone matrix comprises a complex mixture of different proteins that determine its structural integrity and function. Collagen, accounting for approximately 90% of the extracellular matrix (ECM) proteins in primary bone, primarily consists of type I collagen (90%) and non-collagenous proteins (10%) [29,30]. Collagen types II and X are present in the growth plate bone, and they are proposed to participate in bone formation, but they are mainly found in cartilage [31,32]. Mn plays a critical role in forming cartilage and collagen, as well as in bone mineralization [33]. In the process of bone mineralization, Mn can be taken up by osteoblasts. In addition, Mn stimulates the growth of osteoblasts [34].

**Figure 2:**
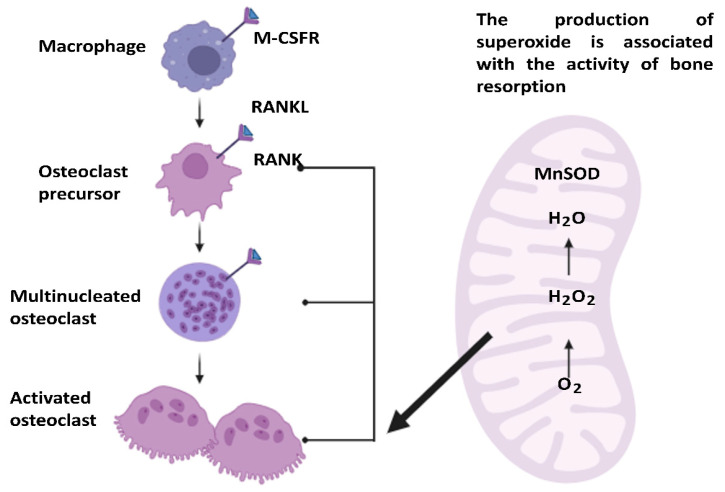
Manganese (Mn) superoxide dismutase (MnSOD) in the bone resorption [52]. RANKL-induced differentiation of macrophages into osteoclasts and the role of MnSOD in managing oxidative stress during bone resorption are depicted. RANKL binds to the RANK receptors on these cells, promoting their maturation. During bone resorption, superoxide (O_2_^−^) is produced as a byproduct, and the mitochondrial enzyme MnSOD catalyzes the conversion of O_2_^−^ into hydrogen peroxide (H_2_O_2_) and oxygen (O_2_), thereby reducing oxidative stress. H_2_O_2_ is subsequently converted into water (H_2_O), providing cellular protection. This process underscores the critical role of MnSOD in maintaining the functionality and integrity of osteoclasts during bone resorption.

Osteoblasts, being active bone-forming cells, are crucial for new bone deposition and mineralization throughout skeletal development and remodeling by regulating osteoclastogenesis and bone resorption [35,36]. Osteoclasts are responsible for bone resorption [37]; another important role of Mn is that these osteoclasts are affected by the main antioxidant enzyme MnSOD [38] (Figure 2).

MnSOD is located in the bone mitochondria [39]. MnSOD plays a role in the differentiation, formation and function of osteoclasts [40]. Due to its localization in the rough boundary membrane of the osteoclast, the production of O_2_^−^ is linked to bone resorption activity. Accordingly, this plasma membrane MnSOD may help protect osteoclasts from extracellular O_2_^−^ anions released during bone resorption [41]. Furthermore, MnSOD catalyzes the formation of hydrogen peroxide (H_2_O_2_) from O_2_^−^, and H_2_O_2_ can stimulate osteoclast differentiation, thereby enhancing MnSOD’s cytoprotective functions against oxidative damage [42]. Reactive oxygen species (ROSs) such as H_2_O_2_ and O_2_^−^ are crucial in osteoclast differentiation, functioning as intracellular signaling molecules [43].

Many signaling pathways are involved in the ROS-mediated modeling of bone mass [44]. Bone mass reflects the balance between bone formation and resorption, which involves the coordinated regulation of osteoblast and osteoclast numbers and activity at the cellular level [45]. At the cellular level, the receptor activator of NFkB (RANK), the receptor activator of nuclear factor kappa beta (NFkB ligand) RANK ligand (RANKL) [46], and osteoprotegerin (OPG) are key components of this signaling system, which regulate bone formation and resorption [47]. The expression of RANKL and OPG regulates osteoclast recruitment and activity by osteoblasts [48]. A member of the tumor necrosis factor (TNF) family, 2–10 RANKL, expressed on the surface of osteoblast/stromal cells, stimulates osteoclastogenesis and the macrophage-colony stimulating factor (M-CSF) in hematopoietic progenitor cells by binding to its surface receptor RANK [49]. In addition, OPG functions as a pseudo-receptor that binds RANKL, acting as a secreted glycoprotein of the TNF receptor superfamily. It does not signal, thereby preventing RANK activation [50]. Through such communication, the RANK/RANKL/OPG axis links osteoblast and osteoclast activity, providing a means of controlling the balance during bone formation and resorption [51]. Through this mechanism, Mn and its enzyme MnSOD play an essential role in the formation of bone mass.

## 4. The Role of Mn in Bone Development and Remodeling

**Figure 3:**
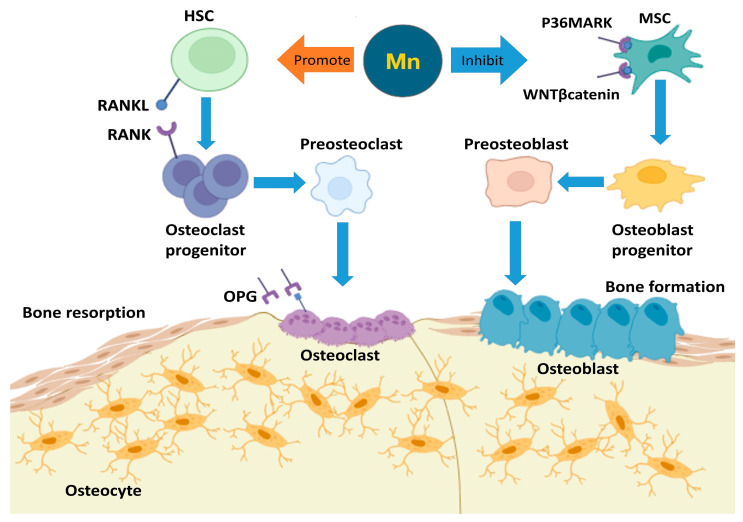
Manganese (Mn) and bone remodeling [72]. The role of Mn in bone remodeling highlights its dual impact on osteoclasts and osteoblasts. Mn promotes osteoclast differentiation by enhancing the RANKL/RANK signaling pathway, where RANKL binds to RANK receptors on osteoclast progenitor cells, leading to their maturation into osteoclasts. Mature osteoclasts resorb bone, a process associated with oxidative stress, during which O_2_^−^ is converted into less harmful molecules by the mitochondrial enzyme Mn superoxide dismutase (MnSOD). Concurrently, Mn inhibits the PI3K/AKT and WNT/β-catenin signaling pathways in mesenchymal stem cells (MSCs), thereby reducing the differentiation and activity of osteoblasts. This dual mechanism underscores the essential role of Mn in maintaining bone mass and integrity, ensuring effective bone regeneration and homeostasis by balancing bone resorption and formation.

Mn is also necessary for the regulation of bone development and bone remodeling [34] (Figure 3). Bone remodeling involves the replacement of old and damaged bone with new bone by a series of cellular events that occur without changing the shape of the bone on one surface [53]. The bone surface consists of osteoclasts and osteoblasts [54], and the main cells are the basic multicellular unit, which are initially responsible for bone remodeling [55]. Bone remodeling is crucial in adult bone homeostasis, and consists of two phases in balance that support bone mass and systemic mineral homeostasis: bone formation and resorption [56]. For the reaction of bone formation and bone resorption, the main basic multicellular unit is a special active structure formed in the process of bone remodeling, covered with a membrane-like structure consisting of bone-resident macrophages (called osteomacs), osteoblasts and osteoclasts, which, acting through the joint, line the bone surface. The process then stops when bone homeostasis is restored [57]. Bone homeostasis is maintained through the balance of osteoblasts and osteoclasts. Bone-resorbing osteoclasts and bone-forming osteoblasts, which are multinucleated giant cells derived from the monocyte/macrophage hematopoietic stem-cell clone, interact to regulate bone mass homeostasis in remodeling processes [58]. 

For bone remodeling one of the traditional bone cells is the osteocyte [59]. Osteocytes are the most abundant and long-living cells in bone, serving as key regulators of bone remodeling [60]. They sense mechanical stimuli through cell bodies, dendrites, and cilia, and can transmit signals via cell dendrites and secreted proteins in an autocrine and paracrine manner [61]. Mechanosensory osteocytes, in the regulation of bone homeostasis, first feel an increase in fluid flow from mechanical loading in the system of lacunar tubules, while both the bone cavity and the endosteal surface are strained, and the cells located there sense intramedullary pressure [62]. Osteocytes are key cells that mediate mechanically induced bone formation and adaptation, as well as inactivity-induced bone loss and skeletal fragility, sensing the mechanical signals responsible for force adaptation to mechanical stimuli [63].

In the remodeling process of bone, the cellular signaling activities are affected by fluid flow; in addition, according to research, the anabolic action of osteoblasts can influence bone regeneration through indirect bone remodeling by mechanically stimulating osteocytes, by activating phosphoinositide 3-kinase/protein kinase B (PI3K/AKT) and the Wingless-related integration site (WNT) signaling pathways [64,65]. The WNT signaling pathway plays a crucial role in bone development and remodeling. It stimulates osteoblast differentiation, proliferation, maturation, and activity [66]. According to the nature of the ligands and the sequence of downstream events, WNT signaling pathways are classified into major canonical WNT signaling and non-canonical WNT signaling pathways, including the canonical WNT pathway, which, in addition to its dependence on the intracellular levels of β-catenin, mainly regulates the differentiation, proliferation and metabolism of osteoblasts, and bone mineralization, which participates in the modulation of its formation [67].

Studies have shown that Mn^2+^ ions activate and stimulate integrins, promoting osteoblast adhesion, viability and proliferation [38]. In addition, recent research suggests that Mn^2+^ ions stimulate osteogenic differentiation and can enhance bone regeneration while preserving bone mass [68]. Furthermore, in the process of bone development and remodeling [69], Mn an essential trace element, necessary for protein synthesis in bone tissue [24]. A number of studies have observed an increase in osteogenic gene expression and an increase in collagen deposition rate in multifunctional materials containing Mn^2+^ ion [70]. Moreover, Mn effects the bone remodeling hormones and the products of the enzymes involved in bone metabolism, with its activity linked to bone [71].

## 5. Mn Absorption and Distribution in the Body

**Figure 4:**
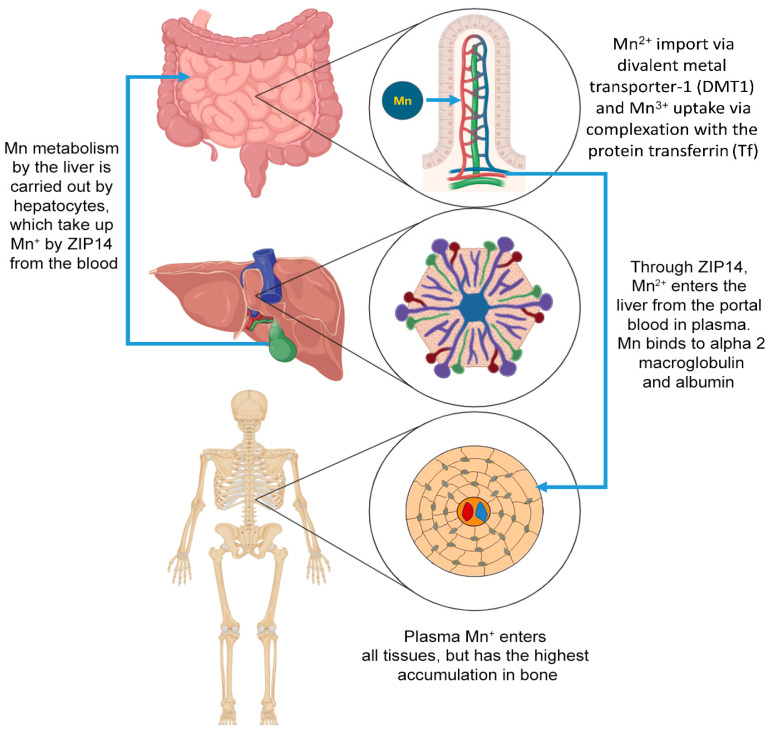
Molecular mechanisms of manganese (Mn) metabolism. The molecular pathways involved in Mn metabolism highlight its absorption, transport, and accumulation in the body. Mn ions (Mn^2+^) are absorbed in the intestines through the divalent metal transporter 1 (DMT1). After absorption, Mn^2+^ ions enter the bloodstream and are transported in a complex with proteins. The liver, considered the central organ in Mn metabolism, plays a crucial role in processing and regulating Mn levels. Mn is then distributed from the liver to various tissues throughout the body, with a significant accumulation in the bones. This high accumulation in bones underscores the essential role of Mn in skeletal health.

Humans consume Mn through dietary intake, with approximately 3–5% of ingested Mn absorbed through the intestinal wall [73] and the remainder excreted in the feces, maintaining a strict homeostatic control of Mn absorption [74]. Dietary Mn, primarily found in the form of Mn^2+^ in the small intestine, is absorbed by molecular mechanisms [75] (Figure 4). Mn in the diet is absorbed throughout the body through the epithelial cells that line the gastrointestinal tract, and enters the bloodstream for tissue use. However, the bulk of Mn absorption is carried out in the small intestine [76]. The current model of Mn metabolism by the liver is through hepatocytes, which take up Mn from blood at the basolateral surface and excrete it into the bile at the apical surface. ZIP14, a close family member of ZIP8, may be responsible for the uptake of Mn from blood, as it has been reported to be localized on the basolateral membrane of hepatocytes [77]. Both the large and small intestines are lined with single-layer protein-binding epithelial cells, with a portion of the cell membrane of each cell in contact with the lumen of the intestinal epithelial cells. This is the apical surface of the cell, where the apical membrane folds into many small folds and emerges into the lumen. This is also called microbubbles, and the edge of the folds greatly increases the available surface area for absorption. 

In addition, a number of absorption mechanisms have been identified on the surface of enterocytes. These mechanisms include Mn^2+^ import via divalent metal transporter 1 (DMT1) and Mn^3+^ uptake via complexation with the protein Tf [78]. After entering the blood, Mn is distributed throughout the body [79]. Various transporters regulate Mn import, including DMT1 or Tf-Tf receptors, the choline transporter, the citrate transporter, and voltage-gated and store-operated calcium channels [80]. Two distinct but related mechanisms responsible for Mn transport are the Tf-dependent and the Tf-independent pathway [81]. In the Tf-dependent pathway, the Tf-Mn^3+^ complex binds to the Tf receptor (TfR) on the cell surface. Endosomal vesicles form on the cell surface after Tf binds to TfR and are subsequently acidified by the hydrogen ion ATPase pump, releasing the metal from the Tf/TfR complex [82]. Mn^2+^ is transported across the endosomal membrane by the transport protein, divalent metal transporter 1 (DMT1; also called natural resistance-associated macrophage protein 2 (Nramp2), divalent cation transporter 1 (DCT1), or solute carrier family 11 member 2 (SLC11A2) [83]. Forming a relatively weak complex of Mn^2+^ with serum albumin (or a2-macroglobulin), the transport of Mn^2+^ released from this complex can be taken directly from the cell surface by DMT1, independent of Tf [84]. From a functional point of view, which of the two uptake mechanisms is dominant may be specific to the cell and depend on the presence of TfR on the cell surface [85]. According to recent data, Mn is absorbed through cell membranes, and the main role of Mn in the regulation of overall cellular function lies in its function as an important cofactor for many enzymes [86].

Also, the water-soluble divalent form of Mn (Mn^2+^) facilitates the transfer divalent metal ions to carriers [87]. DMT1 (divalent metal ion transporter) can also transport Mn^2+^ due to its important role in the intestinal absorption of Mn. The necessity of DMT1 for Mn absorption was shown in the study of intestinal-specific DMT1-null mice [88]. Moreover, among all these proteins, DMT1 is typically the dominant Mn transporter, although it may promote uptake under various physiological or pathological conditions and other transport processes in any known cell population [89].

In recent years, three major Mn transporters have been identified in humans: ZIP8 (SLC39A8), ZIP10 (SLC30A10), and ZIP14 (SLC39A14) [90]. The ZIP8, ZIP10 and ZIP14 are retransmembrane proteins belonging to the Zrt and IRT-like protein (ZIP) family (SLC39 family) [91]. These proteins represent a set of novel molecular mechanisms that control Mn homeostasis [92]. ZIP10, essential for Mn homeostasis in mammals, functions primarily as a physiological Mn transporter rather than a zinc transporter, which is of particular importance in Mn biology [93,94]. 

Also, mainly through ZIP14 in the basolateral membrane of hepatocytes [95], Mn^2+^ enters the liver from the portal blood. In plasma, Mn binds to alpha-2-macroglobulin, albumin, and Tf, or exists as free Mn^2+^ or as Mn^3+^ associated with Tf [95]. Oxidase catalyzes the oxidation of Mn^2+^ to Mn^3+^. Plasma Mn enters all tissues, but the tissue with the highest accumulation is bone tissue, and the accumulation reflects the balance between metal uptake and excretion [96]. Excess Mn in the hepatocyte is excreted into the bile via ZIP10 localized in the apical (canalicular) membrane [73]. Meanwhile, bile containing Mn is excreted into the small intestine, where the Mn is reabsorbed through enterohepatic circulation and eventually excreted in the feces [97].

## 6. Mn Homeostasis and Regulation in Bone Tissue

**Figure 5:**
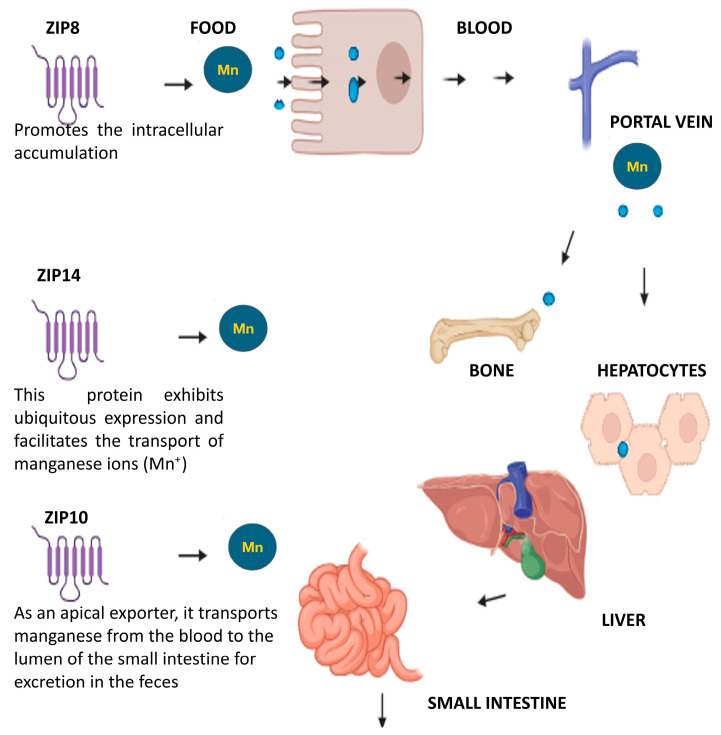
Manganese (Mn) hemostasis in the bone [90]. The cellular mechanisms involved in maintaining Mn homeostasis in bones reveal the key physiological functions of Mn transporters and regulators, including ZIP8, ZNT10, and ZIP14. The process begins with the intake of Mn from food, where ZIP8 facilitates the intracellular accumulation of Mn^2+^ ions. These Mn^2+^ ions enter the bloodstream and are transported to various tissues, including bones and liver hepatocytes. The transport of Mn^2+^ ions into bones and other tissues is facilitated by the ubiquitously expressed ZIP14. Mn^2+^ ions reach the liver, where they undergo further processing and regulation. The ZIP10 transporter acts as an apical exporter, transporting Mn from the blood to the lumen of the small intestine for excretion in feces. These intricate regulatory mechanisms ensure the balance of Mn, which is crucial for maintaining bone health and overall metabolic homeostasis.

As transmembrane proteins, ZIP14 and ZIP8 mediate the cellular uptake of Mn ions [98] (Figure 5). Both ZIP8 and ZIP14 have a high affinity for Mn hemostasis. The key role of ZIP14 in bone has been confirmed by its abundant expression in chondrocytes, the cartilage cells of the growth plate, during bone elongation [99]. The bones grow in length on the epiphyseal plate due to the elongation of the process bone, which is similar to endochondral ossification [100]. The transport of Mn across cell and intracellular membranes is critical for maintaining Mn homeostasis [101]. This implies that protein transporters, which regulate Mn homeostasis, may significantly influence the physiological and pathological processes in bones, involving transporter genes [92,102].

Mn homeostasis in the bone is regulated at the cellular level by various Mn transporters and regulators [103], including ZIP8, ZNT10 and ZIP14. These transporters are also crucial in the regulation of Mn metabolism in bone [102,104]. ZIP8 and ZIP14 are members of the ZIP family, while ZNT10 belongs to the zinc transporter protein (ZNT) family [105]. ZIP family proteins control the flow of metal from the extracellular fluid to the cell cytoplasm [106]. Within the cytoplasm and nucleus, Mn accumulates, leading to notable intracellular redistribution [107]. Mn^2+^ uptake, mediated by ZIP8, is an endogenous function that moves this cation into the cell [108]. The membrane localization of the ZIP8 transporter protein is known to be the plasma membrane [109], surrounding intracellular organelles [110], the Golgi body [111,112,113,114,115], the lysosomal membrane [112], the endoplasmic reticulum [113], and the mitochondrial membrane [114]. ZIP8 is involved in key cellular processes, including cell morphology, adhesion, migration, and cell proliferation [115]. Additionally, ZIP8 regulates cytoskeleton positioning, distribution, and migration [110] and is proposed to regulate Mn metabolism in the tissues, including bone [111]. An overexpression of ZIP8 stimulates the intracellular accumulation of Mn [108].

Bones store the largest amount of Mn in the human body, and Mn in bone has a long half-life (skeletal bone—8.5 years). Therefore, as Mn accumulates in bone, it can serve as a biomarker of long-term exposure [116].

ZIP14 as an essential component of Mn regulation and homeostasis [117]. ZIP14 is localized to cell membranes, wherein it is ubiquitously expressed and transports Mn [118]. In skeletal tissues, ZIP14 is expressed in growth plate chondrocytes, and regulates their differentiation [119]. In bone tissue, ZIP14 is expressed in normal osteoclasts [120]. ZIP14 has been shown to regulate bone homeostasis by influencing osteoclast-mediated resorption [121], and Mn has its own place in bone homeostasis.

## 7. Mechanisms of Mn Action on Bone Cells

Mn acts in the proliferation and differentiation of osteoblastic cells. The effect of Mn on bone metabolism is investigated in osteoblastic MC3T3-E1 cells (the osteoblastic cell line MC3T3-E1 has been established from a C57BL/6 mouse calvaria). The proliferation of cells was stimulated by the presence of an Mn-based compound, Mn-doped β-tricalcium phosphate (MnTCP). Mn0.5-1TCP powder significantly increased MC3T3 cellular proliferation. To assess the biological potential of Mn-doped β-TCP materials for repairing or regenerating bones affected by osteoporosis or severe injuries, various biological responses were evaluated, including cell viability and type I collagen secretion [122]. The Mn compound has demonstrated a stimulatory effect on the cell differentiation of osteoblastic cells [123]. MnCl2 was shown to accelerated osteogenesis by promoting early angiogenesis [124]. Mn-containing BGs exhibited no cytotoxic effects on human mesenchymal stem cells, and an enhanced osteogenic differentiation and mineralization process was confirmed by the high expression of osteogenic differentiation markers, such as alkaline phosphatase (ALP) activity and collagen type I, osteopontin and osteocalcin [33]. In addition, the process of preparing Mn-containing coatings by micro-arc oxidation and plasma immersion ion implantation and deposition (PIIID) can promote collagen secretion and the mineralization of the ECM, showing excellent corrosion resistance properties, which can improve bone differentiation [125].

Mn has an inhibitory effect on osteoclast-like cell formation. Bone-resorbing cells, osteoclasts, are formed by the differentiation of bone marrow cells. In this study, the effects of MnTBAP on osteoclastogenesis, a cell-permeable SOD2 mimetic, were evaluated, and osteoclast formation from BMMs was reduced by MnTBAP in a dose-dependent manner. It also effectively downregulated the expression of osteoclast marker genes and inhibited the induction of nuclear factor of activated T cells (NFATc1) [40]. Furthermore, both in vitro and in vivo results indicate that the Mn^2+^ ions released from Mn-TCP bioceramics can inhibit the formation and function of osteoclasts, promote osteoblast differentiation, and accelerate bone regeneration under osteoporotic conditions. Mechanically, Mn-TCP bioceramics inhibit osteoclastogenesis and activate Nrf2, promoting the repair of osteoporotic bone defects by scavenging ROS [38]. The effect of Mn(II)-enriched C. glomerata methanolic extract on the mRNA expression of osteoclast-related genes in lipopolysaccharide-induced cryopreserved cell lines, derived from Mouse C57BL/6 calvaria (LPS-induced MC3T3-E1) cells, suggests that this extract may attenuate signaling pathways that initiate aberrant osteoclastogenic differentiation [126]. Also, this research demonstrates that Mn^2+^ in composite hydrogels can reduce the ROS level by activating the nuclear factor erythroid 2-related factor 2 (Nrf2) pathway, and fibroblast activation protein inhibitor (FAPi) can inhibit NF-κB signaling pathway. Together, these two mechanisms may contribute to the suppression of osteoclastogenesis and inhibit osteoclast formation by hydrogels [127]. These findings suggest that Mn inhibits osteoclast formation and function (Table 1).

## 8. Mn Deficiency and Its Impact on Bone Health

Mn deficiency may play a pathophysiological role in the deterioration of bone metabolism. Mn deficiency disrupts the balance between osteoblastic and osteoclastic activities. Several studies on rats with low-Mn diets have suggested that such an imbalance inhibits cartilage formation and induces osteopenia [128]. Additionally, it has been shown that Mn deficiency negatively affects the development of chondrocytes by inhibiting their proliferation and inducing apoptosis [129] (Table 2).

This study confirmed that Mn deficiency has the potential to affect tibia development in broiler chickens and is associated with decreased OPG/receptor activator of nuclear factor κB ligand (RANKL) mRNA expression, leading to metaphyseal osteoporosis [130]. The RANKL is a crucial cytokine regulating osteoclast differentiation and survival. Also, studies have shown that Mn deficiency causes avian tibial dyschondroplasia by inhibiting chondrocyte proliferation and differentiation in broilers [131]. According to this research, dietary Mn deficiency impairs serum bone regulatory hormones and bone metabolism enzymes in chicks [132]. In addition to finding that Mn deficiency decreases tibial length and tibial growth plate thickness in a tibial dyschondroplasia model, this study also found that Mn deficiency is irregular under the tibial growth plate, and increases the lesion area tibial dyschondroplasia lesions of white tibial dyschondroplasia [133] (Table 2).

## 9. Mn Toxicity and Its Effects on Bone Mass

Mn is essential for various biological and physiological processes, including bone growth. Bone tissue is the primary organ for Mn accumulation. Long-term excessive exposures to Mn can negatively affect bone metabolism. The mechanisms of Mn toxicity are complex and not fully understood, and bone retains Mn for many years after the initial exposure. Evidence from animal studies shows that Mn has an average elimination half-life in rat skeletal bones of 143 days, which is equivalent to its accumulation in human bones of about 8.5 years. Plasma Mn concentrations in rats in the study were ∼20 μg/L on day 30 after this drinking regimen, which is comparable to human blood Mn levels during poisoning [134].

Based on this study, Mn was reported to accumulate in bone tissue in a population of 60 Chinese industrial workers. This study used neutron activation to measure bone Mn in vivo and characterized occupational Mn exposure using the cumulative exposure index (CEI) to classify occupational exposure into high, moderate, or low-quality exposure ratings based on worker questionnaire responses. The CEI method is used in occupational studies, where there are significant difficulties in performing comprehensive exposure assessments over months and years, but it may not accurately distinguish whether high levels of bone Mn reflect Mn accumulation from long-term exposure (e.g., years to decades) to recent high exposures over a short time, proximal to bone Mn measurements. There is also some evidence that in this study the route of exposure was inhalational, whereas in another study it was oral, and that the effects of Mn have different toxicokinetic profiles following inhalational and oral exposures [135]. At the same time, this study found that retired women in the highest Mn exposure sample (Mn-CEI triple 3) have a higher risk of developing osteoporosis compared to control women. Further research is necessary to explore the potential mechanisms underline changes in bone quality [136]. This study also reported that Mn exposure might decrease bone mineral density, and found a negative association between blood Mn levels and bone mineral density in adolescents, particularly in girls aged 12–15 years. Therefore, elevated blood Mn levels may pose a risk factor for low bone mass in adolescents [137].

## 10. Animal Studies Investigating the Relationship between Mn and Bone Density

Mn is an essential element for bone growth. Several animal studies examined the relationship between Mn levels and bone density. Controlled experiments using various animal models have suggested a possible link between Mn intake and its effects on bone health. One of the studies proposes that, in addition to the therapeutic effects of Mn treatment of colonic inflammation, Mn also modulates bone homeostasis in young Wistar rats with ulcerative colitis [138]. In this research, the chronic oral administration of Mn to animals was conducted, characterizing the time-dependent accumulation of Mn in rat bones and determining the t1/2 of Mn elimination in bone tissues. Bone samples were collected from different parts of the body to investigate whether they exhibit similar or different kinetic characteristics and Mn neurotoxicity in the brain. They found a correlation between Mn concentrations in bone and Mn concentrations in selected regions [134]. In addition, by adding Mn to PDA, the surface characteristics of the Mn-modified peek implant (PEEK-PDA-Mn) group, its roughness and hydrophilicity, and biocompatibility were significantly improved. The expression results of osteogenic genes, ALP and mineralization indicated that Mn immobilization enhances the differentiation capacity of MC3T3-E1 cells into osteoblasts in vitro [139]. This study demonstrated that teeth are a sensitive biomarker of active and past Mn exposure and tissue Mn burden [140]. Animal studies further clarify the effects of Mn exposure on bone throughout the lifespan and suggest potential benefits of using bone as a biomarker for Mn exposure [116]. Additionally, research findings on nitrogen nickel-free stainless steel implantation in rabbit tibiae revealed that nitrogen nickel-free stainless steel induces the osteogenic differentiation of rat bone marrow mesenchymal stem cells and promotes rapid and long-term osseointegration of implants, likely due to the combined effects of Mn and N elements [141]. The study also suggested that the effects of Mn^2+^ on bone marrow mesenchymal stem cells proliferation, osteogenic differentiation, and adipogenic differentiation are complex, with concentration and incubation time being critical factors in modulating the biological effects of Mn^2+^ from injury to protection [142].

## 11. Clinical Studies Assessing Mn Supplementation and Bone Health

Mn is an indispensable trace element necessary for the normal development and activity of tissues such as bones. This study focused on the effects of Mn supplementation on growth, blood biochemistry, nitrogen metabolism, and skeletal development in growing Rex rabbits. As a result of the study, it was found that dietary Mn has a positive effect on the growth rate of Rex rabbits. At the same time, it was observed that Mn supplementation increased nitrogen utilization and decreased serum triglyceride levels. Also, a Mn supplement of 20 mg/kg was found to be the most suitable for improving the growth performance of rabbits [143]. In addition, this study reported that ALP and tartrate-resistant acid phosphatase were evaluated in the tibia and serum of broilers, fed diets with varying levels of phytase and levels of zinc, Mn, and copper. In an experiment involving 1200 male Cobb broilers, reared according to standard commercial breeding methods, it was found that supplements containing zinc, Mn, and copper significantly benefited bone metabolism, as a result of which, among others, Mn increased the activity of the growth plate, accelerated calcification, and contributed to the reconstruction of newly formed tissue into trabecular bone [144]. In a separate study, the effect of an optimized dietary supplement with Mn on growth performance, tibia characteristics, immune function and meat quality of yellow feather broilers were evaluated, and in the diets of birds with an Mn-added optimal mixture, in accordance with nutritional standards, the best performance was achieved [145]. Additionally, organic Mn supplementation is more bioavailable than inorganic Mn; the use of Mn proteinate, especially, has proven to improve leg development and absorption efficiency, as well as general oxidative stress status in broilers [146]. Viegas et al. reported that the addition of high dietary levels of Mn (90 mg kg- and Zn 130 mg kg^−1^ to a commercial microdiet for marine fish larvae improved larval survival and reduced vertebral defects, and increased bone Mn deposition [147]. Furthermore, the addition of Mn-methionine (Mn-Met) to the diet of laying hens affected egg quality, including the internal properties and mechanical properties of the eggshell. As a result, the dietary Mn-Met supplement increased both internal egg quality and eggshell ultrastructure [148]. Venglovska et al. also highlighted the importance of Mn in eggshell formation, noting its positive effect on eggshell quality. [149]. It was also reported that the dietary supplementation of Mn hydroxy chloride can improve broiler antioxidant capacity, bone quality, and Mn deposition [150]. This study showed for the first time that organic Mn supplements, administered to deer fed a balanced diet, improved deer bone quality and some bone mechanical properties [151] (Table 3). 

## 12. Future Directions: Areas for Further Research and Investigation

Numerous studies have shown that adequate levels of Mn can play a role in maintaining optimal bone health. However, the exact mechanisms underlying this relationship between Mn and bone health remain a subject of ongoing research. Further research is needed to fully understand how Mn affects bone growth and bone density to shed light on its potential implications for human skeletal health. These studies provide an important foundation for future research aimed at elucidating the complex interactions between Mn and the regulation of bone health and density. There are several key areas that require further investigation regarding Mn exposure and bone health. These research directions provide a comprehensive analysis of further research in the area of Mn effects on bone health, and studies that monitor the effects of different Mn levels over time in animal models may shed light on long-term effects on bone density and skeletal integrity. Also, studying the specific molecular mechanisms of Mn interaction with bone cells such as osteoblasts and osteoclasts is important to reveal the specific pathways involved in the regulation of bone metabolism. Furthermore, investigating the major role of Mn supplementation or deficiency in bone-related disorders or osteoporosis and skeletal abnormalities may provide valuable clinical research opportunities. Comparative studies across different species and ages may also suggest possible variations in the effects of Mn on bone density, and studying how Mn interacts with other dietary factors to influence bone health may also provide a better understanding of the complex interactions between nutrients and skeletal integrity.

## 13. Conclusions

This comprehensive review underscores the multifaceted role of Mn in bone health, emphasizing its critical influence on bone regeneration, mineralization, and overall skeletal strength. The research findings highlight Mn’s vital role in bone metabolism through its modulation of osteoblast and osteoclast activities, which are essential for bone formation and resorption. Syntheses of experimental models, epidemiological studies, and clinical trials consistently demonstrate Mn’s profound impact on bone mineralization and density, particularly through the regulation of key signaling pathways and enzymatic reactions. Maintaining adequate Mn levels is crucial for optimal bone health, as deficiency is linked to impaired bone growth and an increased risk of bone diseases. Conversely, excessive Mn exposure and accumulation can negatively impact bone metabolism, underscoring the need for balanced intake. Future research should focus on elucidating the specific molecular mechanisms by which Mn influences bone cells and identifying the pathways involved in bone metabolism regulation. Additionally, investigating the role of Mn supplementation in the prevention and treatment of bone diseases, such as osteoporosis, may provide valuable insights into potential therapeutic strategies. This review lays a crucial foundation for future research aimed at elucidating the intricate interactions between Mn and bone health, ultimately contributing to the development of strategies to optimize bone health and prevent skeletal diseases.

## Figures and Tables

**Table 1:** Mechanisms of manganese (Mn) action on bone cells.

Mn Compound Type	Bone Cell Types	Mechanisms of Action	Outcomes	References
Mn compound	Osteoblasts	Proliferation	Evaluated cell viability and type I collagen secretion	[122]
Mn compound	mBMSCs	Differentiation	Effective for osteogenic differentiation	[123]
MnCl_2_	Osteoblasts	Osteogenesis	Accelerated osteogenesis, increased angiogenesis	[124]
Mn containing BG	hMSCs	Differentiation	High expression of osteogenic markers (ALP, collagen type I, osteopontin, osteocalcin)	[33]
Mn-containing coatings by micro-arc oxidation and PIIID	Extracellular matrix	Collagen secretion and mineralization	Improve bone differentiation	[125]
MnTBAP	BMMs	Downregulate osteoclast marker genes	Inhibit induction of NFATc1	[40]
Mn^2+^ ions released from Mn-TCP bioceramics	Osteoclasts	Inhibit osteoclast formation	Decreased osteoclasts, accelerated bone defect regeneration by activating Nrf2 and scavenging ROS	[38]
Mn(II)-enriched C. glomerata methanolic extract	LPS-induced MC3T3-E1 cells	mRNA expression of osteoclast-related genes	Attenuate signaling pathways, reduce aberrant osteoclast differentiation	[126]
Mn^2+^ in composite hydrogels	Osteoclasts	Reduce ROS, inhibit NF-κB signaling	Suppress osteoclastogenesis, inhibit osteoclast formation	[127]

Abbreviations: Plasma immersion ion implantation and deposition (PIII&D), Mn-doped β-tricalcium phosphate (MnTBAP), Mn-contained β-tricalcium phosphate (Mn-TCP bioceramics), bone marrow mesenchymal stem cells (mBMSCs), human mesenchymal stem cells (hMSCs), lipopolysaccharide-induced cryopreserved cell lines derived from Mouse C57BL/6 calvaria (LPS-induced MC3T3-E1 cells), nuclear factor of activated T cells (NFATc), nuclear factor erythroid 2-related factor 2 (Nrf2), reactive oxygen species (ROS), nuclear factor kappa-light-chain-enhancer of activated B cells (NF-κB).

**Table 2:** Effects of manganese (Mn) deficiency on bone health.

Mn Level (mg/kg)	Experimental Subjects	Tissue	Effects of Deficiency	References
Low diet	Rats	Bone	Inhibits cartilage formation and induces osteopenia	[128]
40 mg/kg diet	Chickens	Bone	Inhibits chondrocyte proliferation and stimulates chondrocyte apoptosis	[129]
8.7 mg/kg	Chickens	Bone	Leads to metaphyseal osteoporosis due to decreased OPG/RANKL mRNA expression	[130]
22 mg/kg diet	Chickens	Tibia	Affects chondrocyte proliferation and differentiation in the tibial growth plate	[131]
8.7 mg/kg	Chickens	Serum markers of bone	Causes disorders in bone regulatory hormones and enzymes of bone metabolism in serum	[132]
22 mg/kg	Chickens	Tibial growth plate	HIF-1α up-regulation and autophagy activation protect against Mn deficiency-induced angiogenesis inhibition	[133]

Abbreviations: messenger ribonucleic acid (mRNA), hypoxia-inducible factor-1 α (HIF-1α), tibial dyschondroplasia (TD), tibial growth plate (TGP).

**Table 3:** Supplementation of manganese (Mn).

Types of Supplementations	Species	Outcomes	Target Tissue	Mechanisms	References
Oral	Rex rabbits	Improved growth performance	Skeletal development	Significant effects on bone strength	[143]
Oral	Cobb broilers	Increased growth plate activity, accelerated calcification	Bone tissue	Increased growth plate activity, accelerated calcification	[144]
Oral	Yellow feather broilers	Increased bone density	Tibia characteristics	Increased tibia diameter	[145]
Organic Mn supplementation	Broilers	Improved leg development and absorption efficiency	Tibial bone	Increased growth performance, tibial bone parameters, oxidative stress indicators	[146]
Oral	Marine fish larvae	Increased bone Mn deposition	Bone	Reduced severity of vertebral defects	[147]
Oral, Mn-methionine supplementation	Laying hens	Increased eggshell ultrastructure	Eggshell	Affected egg quality	[148]
Oral, Mn supplementation from its inorganic and organic sources	Laying hens	Improved eggshell quality	Eggshell	Beneficial impact on eggshell quality	[149]
Dietary supplementation with Mn hydroxychloride	Arbor Acres broilers	Increased tibia length, strength, and density index	Tibial bone	Improved antioxidant capacity, bone quality, Mn deposition	[150]
Mn injection	Deer	Increased impact energy and mineral content in antler bone tissue	Bone tissue	Altered mineral composition, improved structure and mechanical properties	[151]

## References

[1] Sromova V., Sobola D., Kaspar P. (2023). A Brief Review of Bone Cell Function and Importance. Cells.

[2] Vanitchanont M., Vallibhakara S.A., Sophonsritsuk A., Vallibhakara O. (2024). Effects of Multispecies Probiotic Supplementation on Serum Bone Turnover Markers in Postmenopausal Women with Osteopenia: A Randomized, Double-Blind, Placebo-Controlled Trial. Nutrients.

[3] Ciosek Z., Kot K., Kosik-Bogacka D., Lanocha-Arendarczyk N., Rotter I. (2021). The Effects of Calcium, Magnesium, Phosphorus, Fluoride, and Lead on Bone Tissue. Biomolecules.

[4] Lopes T.S.B., Shi H., White D., Araujo I.C.S., Kim W.K. (2024). Effects of 25-hydroxycholecalciferol on performance, gut health, and bone quality of broilers fed with reduced calcium and phosphorus diet during Eimeria challenge. Poult. Sci..

[5] Yang X.R., Li L., Nie L.X. (2024). Associations between co-exposure to heavy metals and vertebral compression fracture, as well as femoral neck bone mineral density: A cross-sectional study from NHANES data. PLoS ONE.

[6] Wang C., Zhu Y., Long H.T., Ou M.N., Zhao S.S. (2022). Relationship between blood manganese and bone mineral density and bone mineral content in adults: A population-based cross-sectional study. PLoS ONE.

[7] Wei M., Huang Q., Dai Y., Zhou H., Cui Y., Song W., Di D., Zhang R., Li C., Wang Q. (2022). Manganese, iron, copper, and selenium co-exposure and osteoporosis risk in Chinese adults. J. Trace Elem. Med. Biol..

[8] Mandal S., Kishore V., Bose M., Nandi S.K., Roy M. (2021). In vitro and in vivo degradability, biocompatibility and antimicrobial characteristics of Cu added iron-manganese alloy. J. Mater. Sci. Technol..

[9] Sirri F., Maiorano G., Tavaniello S., Chen J., Petracci M., Meluzzi A. (2016). Effect of different levels of dietary zinc, manganese, and copper from organic or inorganic sources on performance, bacterial chondronecrosis, intramuscular collagen characteristics, and occurrence of meat quality defects of broiler chickens. Poult. Sci..

[10] Von Oettingen W. (1935). Manganese: Its distribution, pharmacology and health hazards. Physiol. Rev..

[11] Christie T. (2007). Mineral Commodity Report 7—Manganese.

[12] Chukanov N.V., Varlamov D.A., Pekov I.V., Zubkova N.V., Kasatkin A.V., Britvin S.N. (2021). Coupled Substitutions in Natural MnO(OH) Polymorphs: Infrared Spectroscopic Investigation. Minerals.

[13] Ghosh S.K. (2020). Diversity in the Family of Manganese Oxides at the Nanoscale: From Fundamentals to Applications. ACS Omega.

[14] Kemmerer A., Elvehjem C., Hart E. (1931). Studies on the relation of manganese to the nutrition of the mouse. J. Biol. Chem..

[15] Wilgus J.H.S., Patton A.R. (1939). Factors Affecting Manganese Utilization in the Chicken. J. Nutr..

[16] Friedman B.J., Freeland-Graves J.H., Bales C.W., Behmardi F., Shorey-Kutschke R.L., Willis R.A., Crosby J.B., Trickett P.C., Houston S.D. (1987). Manganese balance and clinical observations in young men fed a manganese-deficient diet. J. Nutr..

[17] Stern P.H. (1985). Biphasic effects of manganese on hormone-stimulated bone resorption. Endocrinology.

[18] Kehoe R.A., Cholak J., Storey R. (1940). Spectrochemical study of the normal ranges of concentration of certain trace metals in biological materials. J. Nutr..

[19] Chen P., Bornhorst J., Aschner M.A. (2019). Manganese Metabolism in Humans. Postprints Der Universität Potsdam Mathematisch-Naturwissenschaftliche Reihe.

[20] Horning K.J., Caito S.W., Tipps K.G., Bowman A.B., Aschner M. (2015). Manganese Is Essential for Neuronal Health. Annu. Rev. Nutr..

[21] Baj J., Flieger W., Barbachowska A., Kowalska B., Flieger M., Forma A., Teresinski G., Portincasa P., Buszewicz G., Radzikowska-Buchner E. (2023). Consequences of Disturbing Manganese Homeostasis. Int. J. Mol. Sci..

[22] Tinkov A.A., Paoliello M.M.B., Mazilina A.N., Skalny A.V., Martins A.C., Voskresenskaya O.N., Aaseth J., Santamaria A., Notova S.V., Tsatsakis A. (2021). Molecular Targets of Manganese-Induced Neurotoxicity: A Five-Year Update. Int. J. Mol. Sci..

[23] Fernandes J., Hao L., Bijli K.M., Chandler J.D., Orr M., Hu X., Jones D.P., Go Y.M. (2017). From the Cover: Manganese Stimulates Mitochondrial H_2_O_2_ Production in SH-SY5Y Human Neuroblastoma Cells Over Physiologic as well as Toxicologic Range. Toxicol. Sci..

[24] Prasadh S., Gupta M., Wong R. (2022). In vitro cytotoxicity and osteogenic potential of quaternary Mg-2Zn-1Ca/X-Mn alloys for craniofacial reconstruction. Sci. Rep..

[25] Zhao Q., Wu J., Zhang S., Ni X., Wang B., Lu K., Zhang P., Xu R. (2023). Preparation and properties of composite manganese/fluorine coatings on metallic titanium. RSC Adv..

[26] Brotto M., Bonewald L. (2015). Bone and muscle: Interactions beyond mechanical. Bone.

[27] Srinivasan K., Mijares D.Q., Janal M.N., Aranya A.K., Zhang D.S., LeGeros R.Z., Zhang Y. (2020). In vivo efficacy of calcium phosphate-based synthetic-bone-mineral on bone loss resulting from estrogen and mineral deficiencies. J. Biomed. Mater. Res. B Appl. Biomater..

[28] Ma C., Du T., Niu X., Fan Y. (2022). Biomechanics and mechanobiology of the bone matrix. Bone Res..

[29] Carvalho M.S., Cabral J.M.S., da Silva C.L., Vashishth D. (2021). Bone Matrix Non-Collagenous Proteins in Tissue Engineering: Creating New Bone by Mimicking the Extracellular Matrix. Polymers.

[30] Farshidfar N., Tanideh N., Emami Z., Aslani F.S., Sarafraz N., Khodabandeh Z., Zare S., Farshidfar G., Nikoofal-Sahlabadi S., Tayebi L. (2022). Incorporation of curcumin into collagen- multiwalled carbon nanotubes nanocomposite scaffold: An in vitro and in vivo study. J. Mater. Res. Technol.-JmrT.

[31] Cumming M.H., Hall B., Hofman K. (2019). Isolation and Characterisation of Major and Minor Collagens from Hyaline Cartilage of Hoki (*Macruronus novaezelandiae*). Mar. Drugs.

[32] Yang J., Liu Y., He L., Wang Q., Wang L., Yuan T., Xiao Y., Fan Y., Zhang X. (2018). Icariin conjugated hyaluronic acid/collagen hydrogel for osteochondral interface restoration. Acta Biomater..

[33] Barrioni B.R., Norris E., Li S., Naruphontjirakul P., Jones J.R., Pereira M.M. (2019). Osteogenic potential of sol-gel bioactive glasses containing manganese. J. Mater. Sci. Mater. Med..

[34] Miola M., Brovarone C.V., Maina G., Rossi F., Bergandi L., Ghigo D., Saracino S., Maggiora M., Canuto R.A., Muzio G. (2014). In vitro study of manganese-doped bioactive glasses for bone regeneration. Mater. Sci. Eng. C Mater. Biol. Appl..

[35] Plotkin L.I., Bellido T. (2016). Osteocytic signalling pathways as therapeutic targets for bone fragility. Nat. Rev. Endocrinol..

[36] Blair H.C., Larrouture Q.C., Li Y., Lin H., Beer-Stoltz D., Liu L., Tuan R.S., Robinson L.J., Schlesinger P.H., Nelson D.J. (2017). Osteoblast Differentiation and Bone Matrix Formation In Vivo and In Vitro. Tissue Eng. Part B Rev..

[37] Wu Y., Xie L., Wang M., Xiong Q., Guo Y., Liang Y., Li J., Sheng R., Deng P., Wang Y. (2018). Mettl3-mediated m(6)A RNA methylation regulates the fate of bone marrow mesenchymal stem cells and osteoporosis. Nat. Commun..

[38] Li J., Deng C., Liang W., Kang F., Bai Y., Ma B., Wu C., Dong S. (2021). Mn-containing bioceramics inhibit osteoclastogenesis and promote osteoporotic bone regeneration via scavenging ROS. Bioact. Mater..

[39] Ma Z., Sun J., Jiang Q., Zhao Y., Jiang H., Sun P., Feng W. (2024). Identification and analysis of mitochondria-related central genes in steroid-induced osteonecrosis of the femoral head, along with drug prediction. Front. Endocrinol..

[40] Kim H., Lee Y.D., Kim H.J., Lee Z.H., Kim H.H. (2017). SOD2 and Sirt3 Control Osteoclastogenesis by Regulating Mitochondrial ROS. J. Bone Min. Res..

[41] Guo T., Zhang L.Q., Konermann A., Zhou H., Jin F., Liu W.J. (2015). Manganese superoxide dismutase is required to maintain osteoclast differentiation and function under static force. Sci. Rep..

[42] Winterbourn C.C. (2018). Biological production, detection, and fate of hydrogen peroxide. Antioxid. Redox Signal..

[43] Kanzaki H., Shinohara F., Kanako I., Yamaguchi Y., Fukaya S., Miyamoto Y., Wada S., Nakamura Y. (2016). Molecular regulatory mechanisms of osteoclastogenesis through cytoprotective enzymes. Redox Biol..

[44] Li J., Liang J., Wu L., Xu Y., Xiao C., Yang X., Sun R., Zhao J., Xu J., Liu Q. (2022). CYT387, a JAK-Specific Inhibitor Impedes Osteoclast Activity and Oophorectomy-Induced Osteoporosis via Modulating RANKL and ROS Signaling Pathways. Front. Pharmacol..

[45] Chang X., Xu S., Zhang H. (2022). Regulation of bone health through physical exercise: Mechanisms and types. Front. Endocrinol..

[46] Xiong J., Piemontese M., Onal M., Campbell J., Goellner J.J., Dusevich V., Bonewald L., Manolagas S.C., O’Brien C.A. (2015). Osteocytes, not Osteoblasts or Lining Cells, are the Main Source of the RANKL Required for Osteoclast Formation in Remodeling Bone. PLoS ONE.

[47] Ono T., Hayashi M., Sasaki F., Nakashima T. (2020). RANKL biology: Bone metabolism, the immune system, and beyond. Inflamm. Regen..

[48] Marcadet L., Bouredji Z., Argaw A., Frenette J. (2022). The Roles of RANK/RANKL/OPG in Cardiac, Skeletal, and Smooth Muscles in Health and Disease. Front. Cell Dev. Biol..

[49] Marahleh A., Kitaura H., Ohori F., Kishikawa A., Ogawa S., Shen W.R., Qi J., Noguchi T., Nara Y., Mizoguchi I. (2019). TNF-alpha Directly Enhances Osteocyte RANKL Expression and Promotes Osteoclast Formation. Front. Immunol..

[50] Zhang L., Zeng F., Jiang M., Han M., Huang B. (2022). Roles of osteoprotegerin in endocrine and metabolic disorders through receptor activator of nuclear factor kappa-B ligand/receptor activator of nuclear factor kappa-B signaling. Front. Cell Dev. Biol..

[51] Yasuda H. (2021). Discovery of the RANKL/RANK/OPG system. J. Bone Miner. Metab..

[52] Marques-Carvalho A., Kim H.N., Almeida M. (2023). The role of reactive oxygen species in bone cell physiology and pathophysiology. Bone Rep..

[53] Arias C.F., Herrero M.A., Echeverri L.F., Oleaga G.E., Lopez J.M. (2018). Bone remodeling: A tissue-level process emerging from cell-level molecular algorithms. PLoS ONE.

[54] Weivoda M.M., Chew C.K., Monroe D.G., Farr J.N., Atkinson E.J., Geske J.R., Eckhardt B., Thicke B., Ruan M., Tweed A.J. (2020). Identification of osteoclast-osteoblast coupling factors in humans reveals links between bone and energy metabolism. Nat. Commun..

[55] Calvo-Gallego J.L., Manchado-Morales P., Pivonka P., Martinez-Reina J. (2023). Spatio-temporal simulations of bone remodelling using a bone cell population model based on cell availability. Front. Bioeng. Biotechnol..

[56] Krasnova O., Neganova I. (2023). Assembling the Puzzle Pieces. Insights for in Vitro Bone Remodeling. Stem. Cell Rev. Rep..

[57] Lerner U.H., Kindstedt E., Lundberg P. (2019). The critical interplay between bone resorbing and bone forming cells. J. Clin. Periodontol..

[58] Yu W., Zhong L., Yao L., Wei Y., Gui T., Li Z., Kim H., Holdreith N., Jiang X., Tong W. (2021). Bone marrow adipogenic lineage precursors promote osteoclastogenesis in bone remodeling and pathologic bone loss. J. Clin. Investig..

[59] da Silva Mello A.S., dos Santos P.L., Marquesi A., Queiroz T.P., Margonar R., de Souza Faloni A.P. (2016). Some aspects of bone remodeling around dental implants. Rev. Clínica Periodoncia Implantol. Y Rehabil. Oral.

[60] Qin L., He T., Yang D., Wang Y., Li Z., Yan Q., Zhang P., Chen Z., Lin S., Gao H. (2022). Osteocyte beta1 integrin loss causes low bone mass and impairs bone mechanotransduction in mice. J. Orthop. Translat..

[61] Wang Y., Li C.X., Dong H., Yu J.H., Yan Y., Wu X.G., Wang Y.Q., Li P.C., Wei X.C., Chen W.Y. (2022). Mechanosensation of osteocyte with collagen hillocks and primary cilia under pressure and electric field stimulation. Acta Mech. Sinica-Prc..

[62] Choi J.U.A., Kijas A.W., Lauko J., Rowan A.E. (2021). The Mechanosensory Role of Osteocytes and Implications for Bone Health and Disease States. Front. Cell Dev. Biol..

[63] Zeng Y., Riquelme M.A., Hua R., Zhang J., Acosta F.M., Gu S., Jiang J.X. (2022). Mechanosensitive piezo1 calcium channel activates connexin 43 hemichannels through PI3K signaling pathway in bone. Cell Biosci..

[64] Wu V., Helder M.N., Bravenboer N., Ten Bruggenkate C.M., Jin J., Klein-Nulend J., Schulten E. (2019). Bone Tissue Regeneration in the Oral and Maxillofacial Region: A Review on the Application of Stem Cells and New Strategies to Improve Vascularization. Stem. Cells Int..

[65] Wang Y.Q., Wang N.X., Luo Y., Yu C.Y., Xiao J.H. (2020). Ganoderal A effectively induces osteogenic differentiation of human amniotic mesenchymal stem cells via cross-talk between Wnt/beta-catenin and BMP/SMAD signaling pathways. Biomed. Pharmacother..

[66] Li Z., Yuan X., Arioka M., Bahat D., Sun Q., Chen J., Helms J.A. (2021). Pro-osteogenic Effects of WNT in a Mouse Model of Bone Formation Around Femoral Implants. Calcif. Tissue Int..

[67] Miyamoto K., Ohkawara B., Ito M., Masuda A., Hirakawa A., Sakai T., Hiraiwa H., Hamada T., Ishiguro N., Ohno K. (2017). Fluoxetine ameliorates cartilage degradation in osteoarthritis by inhibiting Wnt/beta-catenin signaling. PLoS ONE.

[68] Du M., Liu C.F., Zhang F., Dong W.T., Zhang X.F., Sang Y.H., Wang J.J., Guo Y.G., Liu H., Wang S.H. (2020). Tunable Layered (Na,Mn)V8O20· n H2O Cathode Material for High-Performance Aqueous Zinc Ion Batteries. Adv. Sci..

[69] Lin S., Yang F., Ling M., Fan Y. (2022). Association between bone trace elements and osteoporosis in older adults: A cross-sectional study. Ther. Adv. Musculoskelet. Dis..

[70] Jiang Y., Zhao J., Zhang D. (2024). Manganese Dioxide-Based Nanomaterials for Medical Applications. ACS Biomater. Sci. Eng..

[71] Anish R.J., Mohanan B., Nair A., Radhakrishnan K.V., Rauf A.A. (2024). Protective effect of Pterospermum rubiginosum bark extract on bone mineral density and bone remodelling in estrogen deficient ovariectomized Sprague-Dawley (SD) rats. 3 Biotech.

[72] El-Ganzuri M.A., Ahmed R.R., Bastawy E.M. (2016). Regulatory Mechanisms of Bone Development and Function. Ann. Cytol. Pathol..

[73] Mercadante C.J., Prajapati M., Conboy H.L., Dash M.E., Herrera C., Pettiglio M.A., Cintron-Rivera L., Salesky M.A., Rao D.B., Bartnikas T.B. (2019). Manganese transporter Slc30a10 controls physiological manganese excretion and toxicity. J. Clin. Investig..

[74] Nakata T., Creasey E.A., Kadoki M., Lin H., Selig M.K., Yao J., Lefkovith A., Daly M.J., Graham D.B., Xavier R.J. (2020). A missense variant in SLC39A8 confers risk for Crohn’s disease by disrupting manganese homeostasis and intestinal barrier integrity. Proc. Natl. Acad. Sci. USA.

[75] Gray E.P., Browning C.L., Vaslet C.A., Gion K.D., Green A., Liu M., Kane A.B., Hurt R.H. (2020). Chemical and Colloidal Dynamics of MnO(2) Nanosheets in Biological Media Relevant for Nanosafety Assessment. Small.

[76] Goff J.P. (2018). Invited review: Mineral absorption mechanisms, mineral interactions that affect acid-base and antioxidant status, and diet considerations to improve mineral status. J. Dairy Sci..

[77] Thompson K.J., Hein J., Baez A., Sosa J.C., Wessling-Resnick M. (2018). Manganese transport and toxicity in polarized WIF-B hepatocytes. Am. J. Physiol. Gastrointest. Liver Physiol..

[78] Ye Q., Park J.E., Gugnani K., Betharia S., Pino-Figueroa A., Kim J. (2017). Influence of iron metabolism on manganese transport and toxicity. Metallomics.

[79] Wooten A.L., Aweda T.A., Lewis B.C., Gross R.B., Lapi S.E. (2017). Biodistribution and PET Imaging of pharmacokinetics of manganese in mice using Manganese-52. PLoS ONE.

[80] Peres T.V., Schettinger M.R., Chen P., Carvalho F., Avila D.S., Bowman A.B., Aschner M. (2016). Manganese-induced neurotoxicity: A review of its behavioral consequences and neuroprotective strategies. BMC Pharmacol. Toxicol..

[81] Liu Q., Barker S., Knutson M.D. (2021). Iron and manganese transport in mammalian systems. Biochim. Biophys. Acta Mol. Cell Res..

[82] Aschner M., Costa L.G. (2018). Linking Environmental Exposure to Neurodevelopmental Disorders.

[83] Wolff N.A., Garrick M.D., Zhao L., Garrick L.M., Ghio A.J., Thevenod F. (2018). A role for divalent metal transporter (DMT1) in mitochondrial uptake of iron and manganese. Sci. Rep..

[84] Yanatori I., Kishi F. (2019). DMT1 and iron transport. Free Radic. Biol. Med..

[85] Knutson M.D. (2019). Non-transferrin-bound iron transporters. Free Radic. Biol. Med..

[86] Zhou Q., Fu X., Wang X., Wu Q., Lu Y., Shi J., Klaunig J.E., Zhou S. (2018). Autophagy plays a protective role in Mn-induced toxicity in PC12 cells. Toxicology.

[87] Ray S., Berry S.P., Wilson E.A., Zhang C.H., Shekhar M., Singharoy A., Gaudet R. (2023). High-resolution structures with bound Mn(2+) and Cd(2+) map the metal import pathway in an Nramp transporter. Elife.

[88] Shawki A., Anthony S.R., Nose Y., Engevik M.A., Niespodzany E.J., Barrientos T., Ohrvik H., Worrell R.T., Thiele D.J., Mackenzie B. (2015). Intestinal DMT1 is critical for iron absorption in the mouse but is not required for the absorption of copper or manganese. Am. J. Physiol. Gastrointest. Liver Physiol..

[89] Fujishiro H., Kambe T. (2022). Manganese transport in mammals by zinc transporter family proteins, ZNT and ZIP. J. Pharmacol. Sci..

[90] Winslow J.W.W., Limesand K.H., Zhao N. (2020). The Functions of ZIP8, ZIP14, and ZnT10 in the Regulation of Systemic Manganese Homeostasis. Int. J. Mol. Sci..

[91] Zhang Y., Jiang Y.H., Gao K.F., Sui D.X., Yu P.X., Su M., Wei G.W., Hu J. (2023). Structural insights into the elevator-type transport mechanism of a bacterial ZIP metal transporter. Nat. Commun..

[92] Aydemir T.B., Kim M.H., Kim J., Colon-Perez L.M., Banan G., Mareci T.H., Febo M., Cousins R.J. (2017). Metal Transporter Zip14 (Slc39a14) Deletion in Mice Increases Manganese Deposition and Produces Neurotoxic Signatures and Diminished Motor Activity. J. Neurosci..

[93] Nishito Y., Tsuji N., Fujishiro H., Takeda T.A., Yamazaki T., Teranishi F., Okazaki F., Matsunaga A., Tuschl K., Rao R. (2016). Direct Comparison of Manganese Detoxification/Efflux Proteins and Molecular Characterization of ZnT10 Protein as a Manganese Transporter. J. Biol. Chem..

[94] Zogzas C.E., Aschner M., Mukhopadhyay S. (2016). Structural Elements in the Transmembrane and Cytoplasmic Domains of the Metal Transporter SLC30A10 Are Required for Its Manganese Efflux Activity. J. Biol. Chem..

[95] Hutchens S., Liu C., Jursa T., Shawlot W., Chaffee B.K., Yin W., Gore A.C., Aschner M., Smith D.R., Mukhopadhyay S. (2017). Deficiency in the manganese efflux transporter SLC30A10 induces severe hypothyroidism in mice. J. Biol. Chem..

[96] Haas M., Kočvara M. (2023). Elemental content in the tissues of the song thrush Turdus philomelos I. Accumulation of macro-and microminerals in internal organs and tissues. Oecologia Mont..

[97] Prajapati M., Conboy H.L., Hojyo S., Fukada T., Budnik B., Bartnikas T.B. (2021). Biliary excretion of excess iron in mice requires hepatocyte iron import by Slc39a14. J. Biol. Chem..

[98] Aydemir T.B., Cousins R.J. (2018). The Multiple Faces of the Metal Transporter ZIP14 (SLC39A14). J. Nutr..

[99] Sasaki S., Tsukamoto M., Saito M., Hojyo S., Fukada T., Takami M., Furuichi T. (2018). Disruption of the mouse Slc39a14 gene encoding zinc transporter ZIP 14 is associated with decreased bone mass, likely caused by enhanced bone resorption. FEBS Open Bio..

[100] Lui J.C., Jee Y.H., Garrison P., Iben J.R., Yue S., Ad M., Nguyen Q., Kikani B., Wakabayashi Y., Baron J. (2018). Differential aging of growth plate cartilage underlies differences in bone length and thus helps determine skeletal proportions. PLoS Biol..

[101] Wang Z.Q., Zhang Y.T., Cao C.Y., Liu J.M., Deng Y., Zhang Z.Q., Wang C. (2023). TaNRAMP3 is essential for manganese transport in. Stress Biol..

[102] Huang T., Yan G., Guan M. (2020). Zinc Homeostasis in Bone: Zinc Transporters and Bone Diseases. Int. J. Mol. Sci..

[103] Felber D.M., Wu Y., Zhao N. (2019). Regulation of the Metal Transporters ZIP14 and ZnT10 by Manganese Intake in Mice. Nutrients.

[104] Takagishi T., Hara T., Fukada T. (2017). Recent Advances in the Role of SLC39A/ZIP Zinc Transporters In Vivo. Int. J. Mol. Sci..

[105] Kong N., Zhao Q., Liu C., Li J., Liu Z., Gao L., Wang L., Song L. (2020). The involvement of zinc transporters in the zinc accumulation in the Pacific oyster Crassostrea gigas. Gene.

[106] Wiuf A., Steffen J.H., Becares E.R., Gronberg C., Mahato D.R., Rasmussen S.G.F., Andersson M., Croll T., Gotfryd K., Gourdon P. (2022). The two-domain elevator-type mechanism of zinc-transporting ZIP proteins. Sci. Adv..

[107] Tang H., Li C., Zhang Y., Zheng H., Cheng Y., Zhu J., Chen X., Zhu Z., Piao J.G., Li F. (2020). Targeted Manganese doped silica nano GSH-cleaner for treatment of Liver Cancer by destroying the intracellular redox homeostasis. Theranostics.

[108] Lin W., Vann D.R., Doulias P.T., Wang T., Landesberg G., Li X., Ricciotti E., Scalia R., He M., Hand N.J. (2017). Hepatic metal ion transporter ZIP8 regulates manganese homeostasis and manganese-dependent enzyme activity. J. Clin. Investig..

[109] Coffey R., Knutson M.D. (2017). The plasma membrane metal-ion transporter ZIP14 contributes to nontransferrin-bound iron uptake by human beta-cells. Am. J. Physiol. Cell Physiol..

[110] Nebert D.W., Liu Z. (2019). SLC39A8 gene encoding a metal ion transporter: Discovery and bench to bedside. Hum. Genom..

[111] Kim G., Elnabawi O., Shin D., Pae E.K. (2016). Transient Intermittent Hypoxia Exposure Disrupts Neonatal Bone Strength. Front. Pediatr..

[112] Bin B.H., Bhin J., Seo J., Kim S.Y., Lee E., Park K., Choi D.H., Takagishi T., Hara T., Hwang D. (2017). Requirement of Zinc Transporter SLC39A7/ZIP7 for Dermal Development to Fine-Tune Endoplasmic Reticulum Function by Regulating Protein Disulfide Isomerase. J. Investig. Dermatol..

[113] Fang L., Watkinson M. (2020). Subcellular localised small molecule fluorescent probes to image mobile Zn(2). Chem. Sci..

[114] Riley L.G., Cowley M.J., Gayevskiy V., Roscioli T., Thorburn D.R., Prelog K., Bahlo M., Sue C.M., Balasubramaniam S., Christodoulou J. (2017). A SLC39A8 variant causes manganese deficiency, and glycosylation and mitochondrial disorders. J. Inherit. Metab. Dis..

[115] Geng X., Liu L., Banes-Berceli A., Yang Z., Kang P., Shen J., Tsai K.J., Liu Z. (2018). Role of ZIP8 in regulating cell morphology and NF-kappaB/Snail2 signaling. Metallomics.

[116] Conley T.E., Richardson C., Pacheco J., Dave N., Jursa T., Guazzetti S., Lucchini R.G., Fendorf S., Ritchie R.O., Smith D.R. (2022). Bone manganese is a sensitive biomarker of ongoing elevated manganese exposure, but does not accumulate across the lifespan. Environ. Res..

[117] Zeglam A., Abugrara A., Kabuka M. (2019). Autosomal-recessive iron deficiency anemia, dystonia and hypermanganesemia caused by new variant mutation of the manganese transporter gene SLC39A14. Acta Neurol. Belg..

[118] Morgan S.E., Schroten H., Ishikawa H., Zhao N. (2020). Localization of ZIP14 and ZIP8 in HIBCPP Cells. Brain Sci..

[119] Zhao Y., Cheng C.Q., Wang X.Y., Yuan Z.C., Sun B.B., EL-Newehy M., Abdulhameed M.M., Fang B., Mo X.M. (2024). Aspirin-Loaded Anti-Inflammatory ZnO-SiO2 Aerogel Scaffolds for Bone Regeneration. ACS Appl. Mater. Interfaces.

[120] Hendrickx G., Borra V.M., Steenackers E., Yorgan T.A., Hermans C., Boudin E., Waterval J.J., Jansen I.D.C., Aydemir T.B., Kamerling N. (2018). Conditional mouse models support the role of SLC39A14 (ZIP14) in Hyperostosis Cranialis Interna and in bone homeostasis. PLoS Genet..

[121] Das B.K., Wang L., Fujiwara T., Zhou J., Aykin-Burns N., Krager K.J., Lan R., Mackintosh S.G., Edmondson R., Jennings M.L. (2022). Transferrin receptor 1-mediated iron uptake regulates bone mass in mice via osteoclast mitochondria and cytoskeleton. Elife.

[122] Torres P.M.C., Vieira S.I., Cerqueira A.R., Pina S., Silva O.A.B.D., Abrantes J.C.C., Ferreira J.M.F. (2014). Effects of Mn-doping on the structure and biological properties of β-tricalcium phosphate. J. Inorg. Biochem..

[123] Wu T., Shi H., Liang Y., Lu T., Lin Z., Ye J. (2020). Improving osteogenesis of calcium phosphate bone cement by incorporating with manganese doped beta-tricalcium phosphate. Mater. Sci. Eng. C Mater. Biol. Appl..

[124] Hreha J., Wey A., Cunningham C., Krell E.S., Brietbart E.A., Paglia D.N., Montemurro N.J., Nguyen D.A., Lee Y.J., Komlos D. (2015). Local manganese chloride treatment accelerates fracture healing in a rat model. J. Orthop. Res..

[125] Zhao Q.M., Sun Y.Y., Wu C.S., Yang J., Bao G.F., Cui Z.M. (2020). Enhanced osteogenic activity and antibacterial ability of manganese-titanium dioxide microporous coating on titanium surfaces. Nanotoxicology.

[126] Bourebaba L., Michalak I., Baouche M., Kucharczyk K., Fal A.M., Marycz K. (2020). Cladophora glomerata enriched by biosorption with Mn(II) ions alleviates lipopolysaccharide-induced osteomyelitis-like model in MC3T3-E1, and 4B12 osteoclastogenesis. J. Cell Mol. Med..

[127] Chen Q.X., Li J.Y., Han F., Meng Q.C., Wang H., Qiang W., Li Z.X., Li F.F., Xie E., Qin X.Y. (2022). A Multifunctional Composite Hydrogel That Rescues the ROS Microenvironment and Guides the Immune Response for Repair of Osteoporotic Bone Defects. Adv. Funct. Mater..

[128] Pepa G.D., Brandi M.L. (2016). Microelements for bone boost: The last but not the least. Clin. Cases Miner. Bone Metab..

[129] Wang J., Wang Z.Y., Wang Z.J., Liu R., Liu S.Q., Wang L. (2015). Effects of manganese deficiency on chondrocyte development in tibia growth plate of Arbor Acres chicks. J. Bone Miner. Metab..

[130] Liu R., Jin C., Wang Z., Wang Z., Wang J., Wang L. (2015). Effects of manganese deficiency on the microstructure of proximal tibia and OPG/RANKL gene expression in chicks. Vet. Res. Commun..

[131] Wang C.Y., Xia W.H., Wang L., Wang Z.Y. (2021). Manganese deficiency induces avian tibial dyschondroplasia by inhibiting chondrocyte proliferation and differentiation. Res. Vet. Sci..

[132] Zhaojun W., Lin W., Zhenyong W., Jian W., Ran L. (2013). Effects of manganese deficiency on serum hormones and biochemical markers of bone metabolism in chicks. J. Bone Miner. Metab..

[133] Lu L., Jin C., Dong P.F., Wang Z.Y. (2022). HIF-1alpha upregulation exerts the antagonistic effect against angiogenesis inhibition in manganese deficiency-induced tibial dyschondroplasia of broiler chicks. Vet. Res. Commun..

[134] O’Neal S.L., Hong L., Fu S., Jiang W., Jones A., Nie L.H., Zheng W. (2014). Manganese accumulation in bone following chronic exposure in rats: Steady-state concentration and half-life in bone. Toxicol. Lett..

[135] Rolle-McFarland D., Liu Y., Zhou J., Mostafaei F., Zhou Y., Li Y., Fan Q., Zheng W., Nie L.H., Wells E.M. (2018). Development of a Cumulative Exposure Index (CEI) for Manganese and Comparison with Bone Manganese and Other Biomarkers of Manganese Exposure. Int. J. Environ. Res. Public Health.

[136] Li D., Ge X., Liu Z., Huang L., Zhou Y., Liu P., Qin L., Lin S., Liu C., Hou Q. (2020). Association between long-term occupational manganese exposure and bone quality among retired workers. Environ. Sci. Pollut. Res. Int..

[137] Liu J., Tang Y., Chen Y., Zhang X., Xia Y., Geng B. (2023). Association between blood manganese and bone mineral density in US adolescents. Environ. Sci. Pollut. Res. Int..

[138] Salami A. (2019). Manganese Chloride Attenuates Osteoporosis in Rats with Experimental Ulcerative Colitis. Arch. Basic Appl. Med..

[139] Yang X., Xiong S., Zhou J., Zhang Y., He H., Chen P., Li C., Wang Q., Shao Z., Wang L. (2023). Coating of manganese functional polyetheretherketone implants for osseous interface integration. Front. Bioeng. Biotech..

[140] Austin C., Richardson C., Smith D., Arora M. (2017). Tooth manganese as a biomarker of exposure and body burden in rats. Environ. Res..

[141] Yu Y., Ding T., Xue Y., Sun J. (2016). Osteoinduction and long-term osseointegration promoted by combined effects of nitrogen and manganese elements in high nitrogen nickel-free stainless steel. J. Mater. Chem. B.

[142] Zhang J., Zhang Q., Li S., Hou Y., Zhang H. (2013). The effects of Mn(2+) on the proliferation, osteogenic differentiation and adipogenic differentiation of primary mouse bone marrow stromal cells. Biol. Trace Elem. Res..

[143] Chen X., Yang G., Zhang B., Li F., Liu L., Li F. (2020). Effects of manganese-supplemented diets on growth performance, blood biochemistry, nitrogen metabolism and skeletal development of rex rabbits. J. Trace Elem. Med. Biol..

[144] Santos L.F.J., Goncalves A.M., Neira L.M., Nakagi V.S., Macari M., Laurentiz A.C., Pizauro J.M. (2023). Effects of Supplementation of Zinc, Manganese, or Copper and Different Phytase Levels in Serum and Bone Acid and Alkaline Phosphatases of Broiler Chicks. Braz. J. Poult. Sci.

[145] Wang Y., Gou Z., Lin X., Fan Q., Ye J., Jiang S. (2021). Optimal Level of Supplemental Manganese for Yellow-Feathered Broilers during the Growth Phase. Animals.

[146] Xia W.H., Tang L., Wang Z.Y., Wang L. (2022). Effects of Inorganic and Organic Manganese Supplementation on Growth Performance, Tibia Development, and Oxidative Stress in Broiler Chickens. Biol. Trace Elem. Res..

[147] Viegas M.N., Salgado M.A., Aguiar C., Almeida A., Gavaia P., Dias J. (2021). Effect of Dietary Manganese and Zinc Levels on Growth and Bone Status of Senegalese Sole (*Solea senegalensis*) Post-Larvae. Biol. Trace Elem. Res..

[148] Li L.L., Zhang N.N., Gong Y.J., Zhou M.Y., Zhan H.Q., Zou X.T. (2018). Effects of dietary Mn-methionine supplementation on the egg quality of laying hens. Poult. Sci..

[149] Venglovska K., Grešáková Ľ., Placha I., Ryzner M., Čobanová K. (2014). Effects of feed supplementation with manganese from its different sources on performance and egg parameters of laying hens Original Paper. Czech J. Anim. Sci..

[150] Sun Y., Geng S., Yuan T., Liu Y., Zhang Y., Di Y., Li J., Zhang L. (2021). Effects of Manganese Hydroxychloride on Growth Performance, Antioxidant Capacity, Tibia Parameters and Manganese Deposition of Broilers. Animals.

[151] Cappelli J., Garcia A., Ceacero F., Gomez S., Luna S., Gallego L., Gambin P., Landete-Castillejos T. (2015). Manganese Supplementation in Deer under Balanced Diet Increases Impact Energy and Contents in Minerals of Antler Bone Tissue. PLoS ONE.

